# Multiple Mechanisms Drive the Evolutionary Adaptation of *Phytophthora infestans* Effector Avr1 to Host Resistance

**DOI:** 10.3390/jof7100789

**Published:** 2021-09-23

**Authors:** Lin-Lin Shen, Abdul Waheed, Yan-Ping Wang, Oswald Nkurikiyimfura, Zong-Hua Wang, Li-Na Yang, Jiasui Zhan

**Affiliations:** 1 Key Lab for Biopesticide and Chemical Biology, Fujian Agriculture and Forestry University, Ministry of Education, Fuzhou 350002, China; linlinshen826@163.com (L.-L.S.); waheed90539@gmail.com (A.W.); nk.oswaldo@gmail.com (O.N.); 2 College of Chemistry and Life Sciences, Sichuan Provincial Key Laboratory for Development and Utilization of Characteristic Horticultural Biological Resources, Chengdu Normal University, Chengdu 611130, China; yanpingwang46@126.com; 3 Key Laboratory of Ecological Pest Control for Fujian and Taiwan Crops, Fujian Agriculture and Forestry University, Fuzhou 350002, China; wangzh@fafu.edu.cn; 4 Institute of Oceanography, Minjiang University, Fuzhou 350108, China; 5 Department of Forest Mycology and Plant Pathology, Swedish University of Agricultural Sciences, 75007 Uppsala, Sweden

**Keywords:** effector protein, mutation mechanisms, population genetics, natural selection, oomycete pathogen, intragenic recombination, local adaptation, sustainable disease management

## Abstract

Effectors, a group of small proteins secreted by pathogens, play a central role in antagonistic interactions between plant hosts and pathogens. The evolution of effector genes threatens plant disease management and sustainable food production, but population genetic analyses to understand evolutionary mechanisms of effector genes are limited compared to molecular and functional studies. Here we investigated the evolution of the *Avr1* effector gene from 111 *Phytophthora infestans* isolates collected from six areas covering three potato cropping regions in China using a population genetic approach. High genetic variation of the effector gene resulted from diverse mechanisms including base substitution, pre-termination, intragenic recombination and diversifying selection. Nearly 80% of the 111 sequences had a point mutation in the 512th nucleotide (T512G), which generated a pre-termination stop codon truncating 38 amino acids in the C-terminal, suggesting that the C-terminal may not be essential to ecological and biological functions of *P. infestans.* A significant correlation between the frequency of *Avr1* sequences with the pre-termination and annual mean temperature in the collection sites suggests that thermal heterogeneity might be one of contributors to the diversifying selection, although biological and biochemical mechanisms of the likely thermal adaptation are not known currently. Our results highlight the risk of rapid adaptation of *P. infestans* and possibly other pathogens as well to host resistance, and the application of eco-evolutionary principles is necessary for sustainable disease management in agricultural ecosystems.

## 1. Introduction

Where plants and pathogens are both present, a co-evolutionary process takes place, whereby plants generate a broad array of resistance mechanisms to avoid or mitigate infection by pathogens, and pathogens repeatedly evolve invasive mechanisms to counteract plant defenses [1,2,3]. This “arms race” usually follows the classical gene-for-gene model. The model proposes that pathogen effector genes evolve to escape recognition by receptor proteins that are coded by corresponding host resistance (R) genes, while these host resistance genes evolve constantly to track the changing pathogen elicitors and retain the plant immune response to circumvent pathogen infection [4,5]. In addition to this pivotal function, increasing evidence suggests that effector proteins also play an important role in disease initiation by enhancing host susceptibility and inhibiting host immune systems through manipulating plant cellular and biochemical processes [6,7]. Thus, it is commonly believed that the antagonistic interaction is a main contributor to the high genetic diversity observed in defense genes of plant hosts and pathogenicity genes of pathogens [4].

Over millennia of host–pathogen co-evolution, plants have developed two layers of immune receptors to defend against a diverse array of pathogens. As the first layer of immune response, pattern recognition receptors (PRRs) detect surface-exposed pathogen/microbe-associated molecular patterns (PAMP/MAMPs), leading to PAMP-triggered immunity (PTI) that inhibits pathogen colonization [8,9]. In the second layer of immune response, intracellular resistance (R) proteins detect pathogen-derived effectors, leading to effector-triggered immunity (ETI) that generally causes hypersensitive responses (HR) induced by local cell death [8,9]. Pathogen effector genes play essential roles to evade host defense systems. They are usually located in gene-sparse, transposon-rich regions, i.e., hotspots for creating sequence variations, in pathogen genomes, e.g., References [10,11,12]. More recent studies have discovered that an array of mechanisms including base substitution, deletion, pseudogenization and transcriptional silencing [11,12,13] are involved in the generation of sequence variations in effector genes. This physical location contributes to high genetic polymorphism of effector genes [14,15] and the constant emergence of new pathotypes, leading to quick ‘breakdown’ of host resistance in commercial production [3,16].

Besides mutations, other biological and ecological processes also affect the genetic variation and evolutionary landscape of effector genes. For example, genetic variation and evolution of effector genes could be affected by recombination events directly through the production of new sequences or indirectly through reduced allelic loss due to enhanced effective population size and suppressed hitchhiking selection [15,17,18]. The occurrence and prevalence of plant diseases result from a complex interaction among the host, pathogen and environment (biotic and abiotic). In this context, the genetic variation and evolutionary trajectory of effector genes could also be influenced by ecological factors such as host demography and climatic conditions. Temperature exerts a major effect on all aspects of chemical, biological, ecological and evolutionary processes [19] and may also contribute to the genetic variation and evolutionary trajectory of pathogen effector genes by regulating, for example, their expression, plant population structure, community composition and pathogen life cycle [20,21].

Potato (*Solanum tuberosum* L.) is the fourth-largest food crop in the world [22]. It is ranked after maize, wheat and rice, with an annual global production of >388 million tons and 19 million hectares [23]. *Phytophthora infestans* is the most destructive plant pathogens of agriculture [24,25], causing the late blight disease of potato and tomato. The pathogen can reproduce sexually and asexually and is dispersed locally and internationally by rain splash, air movement and infected plant materials [26,27,28]. Host resistance mediated by R genes is the most effective and environmentally friendly approach to control the potato late blight, but its effectiveness can be quickly rendered due to the evolution of *P. infestans*. Interaction between *P. infestans* and potato can follow the gene-for-gene model, i.e., the potato plant activates a defense response and displays disease-free phenotypes when receptor proteins encoded by resistance genes recognize the effector proteins secreted by *P. infestans.* This incompatible interaction could be changed when the biochemical structure of the effector proteins is modified. For example, only two amino acid substitutions in the *P. infestans* effector *Avr3a* (K^80^I^103^ to E^80^M^103^) are sufficient to prevent the protein from being recognized by the corresponding R3a protein in potato [29]. In *P. infestans*, all effectors have either a conserved RXLR or a conserved Crinkler (CRN) region in the N-terminal that is needed for cross host cell translocation [10,30,31], and their effector functions usually exist in the C-terminal [32]. It is estimated that the *P. infestans* genome hosts ~180 CRN and ~560 RXLR effector genes [10] with more being continuously detected by molecular analyses [6,7,33,34,35]. Like other species, most of these CRN and RXLR genes are located in gene-sparse and repeat-rich regions of the *P. infestans* genome [10].

*Avr1* is an RXLR effector gene of *P. infestans* identified by map-based cloning technology. The corresponding resistance gene (R1) in the potato host originates from *Solanum demissum* [36]. R1 was the first late blight resistance gene cloned [36] and has been commercially used for decades for potato late blight management [36,37,38]. The effector gene encodes a 208 amino acid protein containing a signal peptide, an RXLR domain and a C-terminal region with two W motifs and one Y motif, which interact with the R1 protein to trigger an HR reaction in potato [36]. Further results have shown that R1-mediated immunity is induced inside the nucleus of plant cells and the R1 and *Avr1* interaction is tightly regulated by nucleocytoplasmic transport [39].

Research on effector genes mainly focuses on their molecular and functional dissection [40,41]. The population genetic analysis of effector genes is limited but needed in order to develop sustainable plant disease management, especially given the central role of effector genes in disease epidemiology. Such analyses would lead to understanding what the mechanisms and patterns of evolutionary adaptation of effector genes are, as well as how ecological factors such as local temperature may impact the evolutionary landscape of the genes. These knowledges are highly relevant to public concerns on food production and security under future climatic conditions. In this study, we addressed these questions using *Avr1* sequences generated from >100 isolates of *P. infestans* sampled from different climatic zones in China. The specific aims of this study were as follows: (i) to determine the genetic variation and its spatial distribution of the *Avr1* gene in the *P. infestans* populations sampled from potato fields in China, (ii) to infer genetic and ecological mechanisms generating and preserving the genetic variation of the *Avr1* gene and (iii) to evaluate the potential influence of temperature on the spatial distribution and evolution of the *Avr1* gene.

## 2. Materials and Methods

### 2.1. Pathogen Population

In total, 111 *P. infestans* strains (Table 1), each with a distinct genotype, were included in the current analysis of population genetic structure in the *Avr1* gene. Genotypes of these *P. infestans* strains were previously determined by molecular and phenotypic assays with SSR markers, sequence analysis of functional genes and mating type characterization [42]. The pathogen strains were isolated from infected potato leaves showing typical late blight symptoms (blackish water-soaked lesions on leaves). The leaf samples were taken from commercial fields located in six provinces across the three major potato cropping regions of China between 2010 and 2011, with two provinces (populations) in each cropping region. These were Ningxia and Gansu in the Northern single-Cropping region (NSR), Guizhou and Yunnan in the Southwestern multiple-cropping region (SMR), and Fujian and Guangxi in the Southern winter cropping region (SWR) [43]. The isolates were maintained on rye B media in Petri plates at 13 °C for long-term storage. Detailed information on sampling and pathogen isolation, purification and genotyping can be found in previous publications [43,44].

### 2.2. DNA Extraction and Avr1 Sequencing

*Phytophthora infestans* isolates were cultured on rye B media at 18 °C in the dark for two weeks after they were retrieved from long-term storage. The harvested mycelia were transferred into sterile 2 mL centrifuge tubes, and genomic DNA was extracted using the Plant gDNA Miniprep Kit (GD2611, Biomiga, Shenzhen, China). The resulting DNA was kept at −20 °C until use. PCR amplification of the genomic DNA was achieved by using the primers Avr1F (5′-ATGGGCTTAATGCACCGC-3′) and Avr1R (5′-TTAAAATGGTACCACAACATG-3′). The reaction volume of the PCR amplification was 25 μL, including 2.5 μL 10× Trans TaqHiFi Buffer, 2 μL dNTPs (2.5 mmol·L^−1^, each 1 μL of a forward primer and reverse primer (10 μmol·L^−1^), 0.20 μL TransTaq HiFi Polymerase (5 U·μL^−1^), 1 μL Template DNA and 17.7 μL ddH_2_O. The PCR amplification begun with an initial denaturation at 94 °C for 4 min, followed by 35 cycles of 94 °C of denaturation for 30 sec, 55 °C of annealing for 30 sec, 72 °C of elongation for 1 min and finally an elongation cycle of 72 °C for 5 min. The PCR products were separated by agarose gel electrophoresis and purified with the QIA quick^®^ Gel Extraction Kit according to the manufacturer’s instruction. The target fragments with the expected size were ligated into the T5 zero cloning vector and transformed into Trans1-T1 competent cells by heat shock at 42 °C for 30 s (pEASY^®^-T5Zero Cloning Kit). Six colonies randomly chosen from each transformation were grown in liquid LB media at 37 °C. Colony morphology was used to identify positive transformation events, and one sample was sent for sequencing (Thermo Fisher Scientific, Shanghai, China).

### 2.3. Data Analysis

All nucleotide sequences were visually evaluated to remove potential artifacts [15]. After this assessment, a total of 111 nucleotide sequences were assembled and aligned with the reference sequence (T30-4) downloaded from GenBank to detect the sites and types of mutations using MEGA5 [45]. The DnaSP 5.10 program was used to deduce amino acid isoforms from the nucleotide sequences [46] and estimate population genetic parameters of the *Avr1* gene, such as haplotype diversity, nucleotide and amino acid diversity, as well as single polymorphic sites and overall population differentiation (*G_ST_*). Only the complete sequences, i.e., sequences without pre-termination, were used for the estimate of amino acid diversity. SSR data of the 111 *P. infestans* isolates were taken from previous publications [42], and overall population differentiation in the SSR markers (*F_ST_*) was evaluated by POPGENE 1.32. Natural selection in the pathogen populations was evaluated by a t-test between the genetic differentiation in *Avr1* gene (*G_ST_*) and SSR marker loci (*F_ST_*) as described previously [15,47] using the standard deviation of *F_ST_* generated from 1000 resamples of original SSR data.

Putative recombination sites within the *Avr1* gene and their parental sequences were identified using the seven algorithms (RDP, GENECONV, Bootscan, MaxChi, Chimaera, SiScan and 3Seq) implemented in the RDP4 suite [48]. The detection of putative recombination sites and their parental sequences were corrected by a Bonferroni procedure with a cut-off of *p* < 0.01. Recombination sites and their parental sequences were displayed by similarity plots implemented in SimPlot 3.5.1 [49] with a window size of 20 nucleotides and a step size of two nucleotides. Annual mean temperature at the collection sites estimated from the historical temperature recorded in the past 15–20 years was taken from a previous publication [44]. The impact of local mean temperature on the spatial distribution of *Avr1* sequence with pre-termination stop was evaluated by multiple regressions, with temperature and temperature-squared as the independent variables [50]. Phylogenetic trees were reconstructed from 111 SSR genotypes and *Avr1* sequences using the neighbor-joining method embedded in MEGA 7.0.21 and displayed by an online tool, the Interactive Tree of Life (iTOL) (https://itol.embl.de/ accessed on 4 September 2021).

## 3. Results

### 3.1. Sequence Variation in Avr1

**Figure 1:**
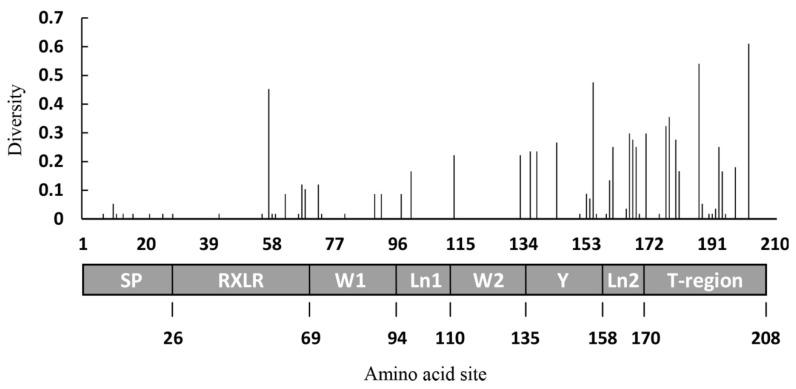
Gene structure and distribution of amino acid diversity in *Avr1*. The amino acid diversity was estimated from *Avr1* sequences without pre-termination. Note: All abbreviations in the grey box indicate different domains/regions of the Avr1 protein.

**Figure 2:**
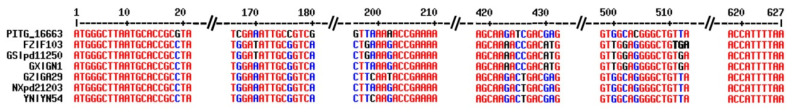
Representative nucleotide haplotypes showing point mutation and pre-termination sites of *Avr1* gene. The name of isolates is presented in the left side of corresponding sequence.

A total of 104 single polymorphic sites were found in the 111 nucleotide sequences, representing 17–20 from each of the six populations (Table 1). These variable sites formed 70 nucleotide haplotypes, corresponding to 49 amino acid isoforms (Table 1, Figure S1). The majority of sequence variations were generated by point mutations. Nearly 39% of the deduced amino acid isoforms differed in only one amino acid residue generated by a non-synonymous mutation. Approximately 73% of the non-synonymous mutations occurred in the effector domain (Figure 1). Amino acid diversity was high in the region after the Y motif, especially in the T-region (Figure 1). Ninety-one isolates from the six populations had a mutation in the 512th nucleotide (T512G), generating a pre-termination stop codon that preictally truncates the entire T-region (38 amino acids) of the *Avr1* gene (Figure 2).

The haplotype diversity of nucleotide sequences in the six populations ranged from 0.889 to 0.984 with a grand mean of 0.975 when all 111 sequences from the six populations were combined. Nucleotide diversity of the six populations ranged from 0.008 to 0.031 with a grand mean of 0.022 (Table 1). The Gansu population displayed the highest haplotype diversity and nucleotide diversity. The lowest haplotype and nucleotide diversity were found in the Fujian population. The overall genetic differentiation across the six populations in Avr1 (*G_ST_*) and SSR marker loci (*F_ST_*) were 0.296 and 0.050, respectively. *G_ST_* was significantly higher than *F_ST_* (*p* < 0.01) by a two-tailed t-test. Further analysis showed that there was no association between the genealogy trees of SSR marker loci and *Avr1* sequences (Figure S2).

### 3.2. Frequency and Spatial Distribution of Avr1

**Figure 3:**
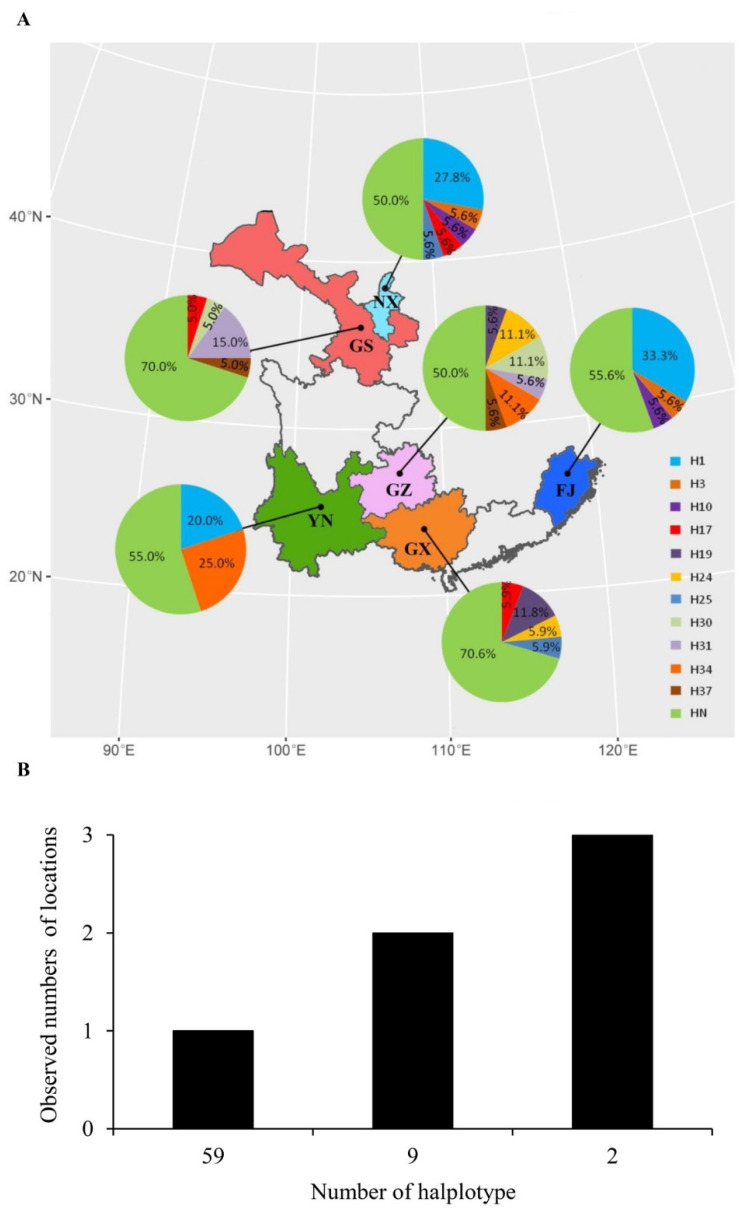
Frequency and spatial distribution of *Avr1* nucleotide haplotypes in the *Phytophthora infestans* populations sampled from six geographic locations of potato fields in China: (**A**) haplotype frequency; (**B**) spatial distribution. Note: HN represents haplotypes detected in only one region.

**Figure 4:**
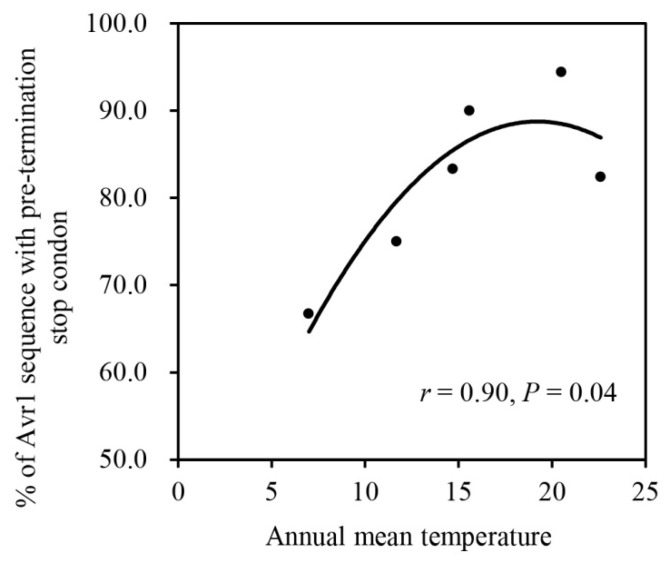
Correlation between the annual mean temperature in the collection sites and the frequency of *Avr1* sequence with pre-termination stop codon.

H1 and H34 were the most common nucleotide haplotypes, accounting for 13.5% and 6.3% of the combined population, respectively. H1 was detected in three populations (Fujian, Guizhou and Yunnan), while H34 was only found in two populations (Guizhou and Yunnan). In addition, H17 was also detected in three populations, and H3, H10, H19, H24, H25, H30, H31 and H37 were each found in two populations (Figure 3). All other haplotypes were detected in only one population (Figure 3). There was a significant correlation between the frequency of *Avr1* sequence with pre-termination stop codon and the annual mean temperature of the six collection sites (r = 0.9, DF = 3, *p* = 0.04, Figure 4).

### 3.3. Intragenic Recombination and Thermal Correlation of Avr1

**Figure 5:**
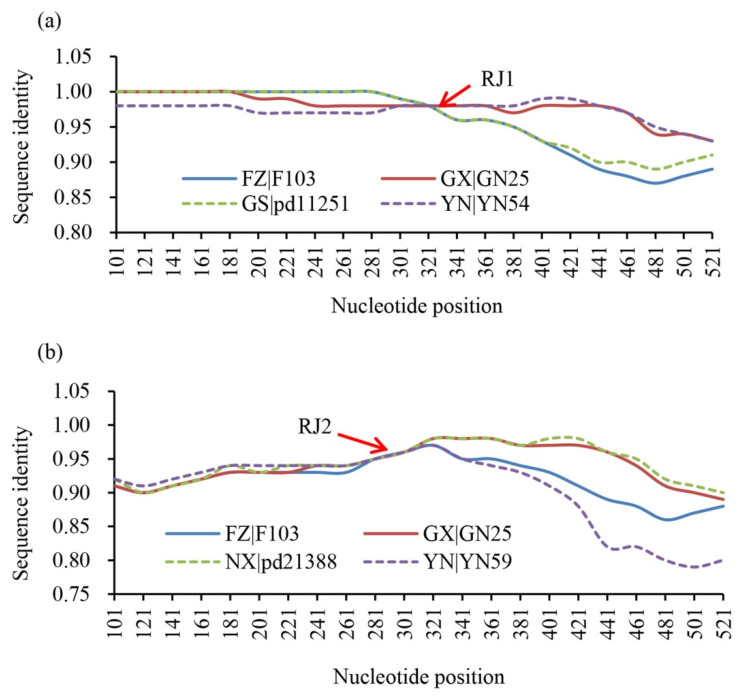
Potential recombination sites and their candidate parental sequences evaluated by Simplot: (**a**) intragenic recombinant generated by F103 and GN25 or pd11252 and YN54; (**b**) intragenic recombinant generated by F103 and GN25 or pd21388 and YN59; RJ1 and RJ2 indicated the putative recombination break points.

Intragenic recombination was detected in one (GN6) of the sequences from the Guangxi population by three of the seven models with high confidence (MAXCHI, *p* = 3.69 × 10^−5^; CHIMAERA, *p* = 4.29 × 10^−6^; and 3SEQ, *p* = 4.4 × 106^−8^). Intragenic recombination was also found in another Guangxi isolate (GN6) by two models and the recombinant was probably generated between F103 and GN25 or pd11251 and YN54 (Figure 5a). Meanwhile, there was another intragenic recombination event found in one (pd11201) of the sequences from the Gansu population. This was also supported by three of the seven models with high confidence levels (MAXCHI, *p* = 6.60 × 10^−3^; SISSCAN, *p* = 9.21 × 10^−7^; and 3SEQ, *p* = 4.64 × 10^−4^) and the recombinant was probably generated between F103 and GN25 or pd21388 and YN59 (Figure 5b).

## 4. Discussion

This is the first attempt to understand the population genetic structure and adaptive mechanisms of the *Avr1* gene in the *P. infestans* using a sequencing approach. This analysis discovered high genetic variation in the effector gene. A total of 70 nucleotide haplotypes with 104 single polymorphic sites were detected in the 111 isolates (Table 1) and nucleotide diversity of the combined population reached 0.022. The level of genetic variation is similar to that detected in effector genes but significantly higher than that detected in other functional genes of many pathogens including *P. infestans* [15,51,52]. For example, using some of the same *P. infestans* isolates, 51 nucleotide haplotypes of the *Avr3a* gene were detected in 96 isolates [15], but only 5 *eEF-la* nucleotide haplotypes were detected in the 165 isolates [53] and 9 RAD23 nucleotide haplotypes were detected in the 140 isolates (unpublished data).

Effector genes contribute to the ability of a pathogen to invade, colonize and reproduce [16,54,55]. The high genetic variation in the effector gene *Avr1* is consistent with an elevated rate of evolution of genes associated with an antagonistic host–pathogen interaction [14,56]. This has been documented in many pathogen species including *P. infestans* [57,58,59]. The high rate of evolution in effector genes such as *Avr1* facilitates the generation of new invasive mechanisms, which allow the pathogen to escape host defenses [16]. This creates an advantage for the survival and reproduction of the pathogen and contributes to the quick breakdown of host resistance in the potato and many other crops [60,61,62,63]. R1 is the first gene introgressed from *S. demissum* to cultivated potato crops for late blight resistance. Widespread commercial use of potato cultivars with the R1 gene [64] creates an environment favoring the evolution of new variants at the *Avr1* site to avoid resistance recognition.

All variations found in this study were generated by base substitution and no deletions or insertions were detected in the sequences, indicating that point mutation is one of main mechanisms driving the evolution of *Avr1*. Interestingly, nearly 82% of 111 sequences had a point mutation in the 512th nucleotide (T512G), which generates a pre-termination stop codon and predictably results a 38 amino acid truncation in the entire T-region of the C-terminal (Figure 1). It has been documented that the truncated effector cannot trigger HR when it is co-expressed with R1 [65]. A combined analysis at an organismal level with published phenotypic data [66] also revealed that the majority of isolates with pre-termination stop codon sequences, which induced late blight symptoms on differential plants containing R1. For example, the *Avr1* sequence with the pre-termination stop codon accounted for 90%, 83% and 67% of Yunnan, Guizhou and Ningxia populations, respectively, which correlates well with the frequency of virulent phenotypes (60%, 55% and 50%) on the R1 differential cultivar [66]. The lower frequency of phenotypes than genotypes in these populations can likely be attributed to the recessive nature of inheritance in virulent mutants. In the sequences with an intact C-terminal (without truncation), most amino acid substitutions occurred between the Y motif and the T-region (Figure 1), the essential site for the interaction of the *Avr1* effector with Sec5 to trigger host cell death [65]. Taken together, these results indicate that the pathogen can tolerate frequent mutations or loss of an entire region without a severe penalty on the fitness of the pathogen. However, although the T512G pre-mature strop codon mutation occurs in the start point of T-region (Figure 1), we realize that the truncation is the theoretical prediction from open-reading frames rather than from empirical data.

Recombination, occurring either within a single gene (intragenic recombination) or between genes (intergenic recombination), can generate both new genotypes through random assortment of existing alleles of different genes and new alleles by rearrangement of nucleotide sequences within the gene [67,68]. It can influence the formation of genetic variation in pathogen populations, even though excessive variation in populations may be deleterious or even lethal [67,68,69,70,71,72,73]. In addition, recombination may also increase genetic variation of pathogen populations by mitigating the variation reduced by hitchhiking selection [15] or enforcing the variation generated by migration [11,27]. Analyses concerning the contribution of intragenic recombination to the adaptive evolution of species are limited relative to intergenic recombination, particularly in the field of plant pathology, but the number has been increasing in the past years due to the increasing application of sequence technology to evolutionary studies and advances in computation tools [15,52,67,68,69,70,72,73]. In this study, we found that intragenic genetic recombination may also act as a contributor to the genetic variation and adaptation of *Avr1* (Figure 5) and the same genetic event was also detected in other *P. infestans* effector genes such as *Avr3a* and *Avr2* [15,52]. *P. infestans* can propagate both sexually and asexually in its life cycle, and sexual oospores serve as a main source of primary inoculums leading to epidemics of late blight in some regions such as Northern Europe [24]. Although A1, A2 and self-fertile mating types have been detected in many parts of China [43], sexual reproduction has not been reported yet in the field populations of the pathogen in the country, suggesting that other channels of genetic exchange such as somatic hybridization may occur in nature there.

In addition to mutation and intragenic recombination, diversifying selection may also contribute to the high genetic variation of the effector gene as supported by significantly higher population differentiation in *Avr1* sequences than that in SSR markers. Genetic differentiation among pathogen populations could result from local adaptation to spatial heterogeneity in selection pressure, such as host resistance and climatic conditions, or from random genetic drift due to finite population size, founder effects or bottlenecks. Genetic drift should affect the entire genome of the pathogen equally [74], leading to a similar extent of population differentiation across the genome, i.e., SSR markers and *Avr1* sequence in our case. On the other hand, natural selection only affects the sequences involved in biological functions and ecological adaptation of species, resulting in different degrees of population differentiation in selected and neutral sequences. Because the SSR marker loci used in the current study are neutral [75], a comparison of *G_ST_* with *F_ST_* can be used to determine whether the *Avr1* sequence is under selection and the type of selection. Diversifying selection for ecological adaptation to local environments increases population differentiation of *Avr1* sequences, generating a significantly higher *G_ST_* among *Avr1* sequences than *F_ST_* from SSR marker loci. On the other hand, constraining selection, occurring when different environments favor similar characteristics among populations inhabiting different ecological niches, would decrease population differentiation of the sequences under selection, leading to a significantly lower *G_ST_* than *F_ST_*. If *G_ST_* from the *Avr1* sequences is not significantly different from the *F_ST_* resulting from the SSR marker loci, the hypothesis of neutral evolution in *Avr1* sequences will be retained [76]. Diversifying selection, driven by ecological heterogeneity, can lead to the preservation of various mutants adapting to a particular environmental condition [77]. In China, host resistance has been a primary method to control potato late blight for decades, and spatial heterogeneity and dynamics in resistance deployment [64] are a likely explanation for the observed diversifying selection.

By regulating survival, reproduction and movement of species, climatic factors such as temperature can also affect the prevalence and evolution of plant pathogens. For example, a persistent increase in the extinction rate of the rust pathogen *T. ulmariae* and a decline in the prevalence and severity in its associated hosts over a 30-year period was found in Northern Sweden, due to the change of local temperature [78]. Adaptive evolution mediated by local thermal conditions has also been documented at an organismal level recently in several plant pathogens including *Zymoseptoria tritici* [79], *Rhynchosporium commune* [80] and *P. infestans* [7]. A significant correlation between the frequency of *Avr1* sequence with pre-termination changes and annual mean temperature in the collection sites (Figure 4) was found in the study, suggesting that thermal heterogeneity might also contribute to the observed diversifying selection of the effector gene. Although we cannot prove the direct link of temperature to the sequence characters by the correlation analysis in the study of the *Avr1* gene, the events of temperature-regulated effector expression, translocation, spatial distribution and interaction with host immunity have been documented in several plant–pathogen interactions [22,81,82] including *Avr2* and *Avr3a* of *P. infestans* [15,52]. Therefore, we believe that the same mechanisms might also be involved in the biological, biochemical and evolutionary process of the effector *Avr1* in *P. infestans*. In future, additional functional analyses should be conducted to confirm the hypothesis.

## 5. Conclusions

In the gene-for-gene model of host–pathogen interaction, plant defenses are triggered by successful host recognition of effector proteins. To evade this recognition, pathogens must quickly and constantly modify the biochemical structure of effector proteins. Previously, we found that *P. infestans* achieves this evasion by mutations that generate the change of an effector from a structured protein to a disordered protein [52]. In this study, we found that *P. infestans* evades host recognition by discarding a part of an effector through a point mutation leading to pre-termination of the translation. The same approach was also used by *Avr4* of the pathogen to escape potato host recognition [83]. These results suggest that *P. infestans*, and perhaps other pathogens as well, have employed an array of mutation mechanisms to adapt to host resistance, threatening plant disease management and sustainable food production. In this case, the application of eco-evolutionary principles to reduce the adaptive potential of pathogens by constraining the movement and population size of plant pathogens and to create divergent selection through spatiotemporal deployment of resistance genes becomes more important for sustainable disease management in agricultural ecosystems [2,3,84].

## Figures and Tables

**Table 1:** Annual mean temperature and summary statistics of genetic diversity in *Avr1* of six *Phytophthora infestans* populations sampled from China.

Population	Location	T^a^	Number of Sequences	Number of Haplotypes	S^b^	O^c^	Haplotype Diversity	Nucleotide Diversity
Ningxia	Guiyuan	7	18	15	47	12	0.974	0.025
Gansu	Tianshui	11.7	20	17	57	14	0.984	0.031
Guizhou	Anshun	14.7	18	13	56	10	0.928	0.019
Yunnan	Kunming	15.6	20	13	58	11	0.916	0.015
Fujian	Fuzhou	20.5	18	11	23	10	0.889	0.008
Guangxi	Nanning	22.6	17	14	69	8	0.978	0.026
Total	/	/	111	70	104	65	0.975	0.022

T^a^ *=* Annual mean temperature in collection sites. S^b^ = Single polymorphic sites. O^c^ = Sequences only found in one place.

## Data Availability

Haplotype data generated in this study are deposited in NCBI with the accession numbers MZ667748–MZ667858.

## Supplementary Materials

The following are available online at https://www.mdpi.com/article/10.3390/jof7100789/s1, Figure S1: List of 70 *Avr1* haplotypes and their corresponding change in amino acid. Figure S2: The Neighbor-Joining phylogenetic trees reconstructed from 111 *Phytophthora infestans* isolates collected from six geographic locations or China: (A) *Avr1* sequences and (B) SSR markers.
